# Supplementary material for: Fast gradient-free optimization of excitations in variational quantum eigensolvers
Source: Commun Phys. 2025 Oct 30;8(1):418. doi: 10.1038/s42005-025-02375-9 (PMC12576945; doi:10.1038/s42005-025-02375-9)
Supplement: Supplementary file 2 — Supplementary Information [file 42005_2025_2375_MOESM2_ESM.pdf]

Supplementary Information for “Fast gradient-free optimization of excitations in variational quantum eigensolvers”

Supplementary Notes Contents

1 Experimental setup **1**
1.1 Hyperparameter tuning and calibration 1
1.2 Fixed UCCSD ansatz 2
1.3 Adaptive Ansatz 2
1.4 ExcitationSolve 2D optimization 3
1.5 Dissociation experiments 3
1.6 Shot noise simulations 3
1.7 NISQ hardware experiments 4

2 Experimental results and analyses **6**
2.1 Hyperparameter tuning results 6
2.2 ADAPT-VQE resource comparison 6
2.3 ADAPT-VQE impact analysis of operator selection criterion vs parameter optimizer . 6
2.4 Dissociation curve energy errors 9
2.5 Analysis of dissociation curve experiments on local minima avoidance 10
2.6 Shot noise 10
2.7 NISQ robustness of ExcitationSolve adaptive operator ranking 12

3 ExcitationSolve algorithmic details **14**
3.1 ExcitationSolve for fixed ansätze 14
3.2 ExcitationSolve for ADAPT-VQE (adaptive ansätze) 15
3.3 ExcitationSolve for multiple occurrences of multiple parameters 15
3.4 Reconstruction strategies for noise robustness 16

4 Proofs **17**
4.1 Analytic energy function in single parameter 17
4.2 $G^3 = G$ and $G^2 \neq I$ for generators of excitation operators 19
4.3 General Fourier Series for Multi-Parameter Optimization 20
4.4 Fourier series for multiple occurrences of a single parameter 22

5 Comprehensive overview of standard approaches in variational quantum algorithms **23**
5.1 Gradients via parameter-shift rules for excitation operators 23
5.2 Quantum-aware optimization for rotations: Rotosolve and SMO 23
5.3 ADAPT-VQE 24

Supplementary Note 1: Experimental setup

The majority of the experiments were implemented in Python using the quantum computing framework `Pennylane` [1].

Supplementary Note 1.1: Hyperparameter tuning and calibration

For both GD and Adam we perform a hyperparameter tuning for the step size. For Adam we set the momentum parameters to $\beta_1 = 0.9$ and $\beta_2 = 0.99$ and keep them fixed for every step size. We consider the following step sizes: 0.5×10^n and 2.5×10^n for $n \in \{-4, -3, -2, -1, 0\}$. Figure S1 in the

Supplementary Note shows the performance of the investigated step sizes. SPSA also has two tunable hyperparameters, the learning rate and the perturbation for the gradient approximation. We use the SPSA implementation in `qiskit` [2] and use its calibration function to tune both hyperparameters before starting the VQE optimization at the constant cost of 50 energy evaluations [3]. To account for this calibration phase, we plot the Hartree-Fock energy for the first 50 SPSA energy evaluations for each molecule. We find that tuning the hyperparameters of COBYLA has negligible impact on the convergence and, therefore, use the default parameters of the `SciPy` implementation [4]. For the BFGS optimizer we used the `SciPy` implementation which has no hyperparameters. We emphasize again that ExcitationSolve has no hyperparameters that need to be tuned.

Supplementary Note 1.2: Fixed UCCSD ansatz

We choose the UCCSD ansatz in its first Trotter-approximation and use the STO-3G basis set as provided by the Pennylane datasets [5] and the Jordan-Wigner (JW) mapping [6]. The single- and double excitations are ordered as provided by Pennylane [1] (version `0.37.0`). The system is initialized in the HF state and zero parameters. In each VQE iteration, all parameters are optimized in the order in which they appear in the ansatz (first the double-, then the single-excitations), unless explicitly stated otherwise. The step sizes for each molecule determined through hyperparameter tuning are listed in Table S1.

Table S1: Optimal step sizes for the GD and Adam optimizers.

	**GD**	**Adam**
H_2	0.5	0.005
$H_3{}^+$	0.5	0.005
LiH	0.25	0.005
H_2O	0.025	0.0025

Supplementary Note 1.3: Adaptive Ansatz

Table S2: Optimal threshold values in Ha for operator selection and VQE convergence. For ExcitationSolve the threshold is an absolute energy difference between evaluations, for GD the threshold is a gradient.

	**ExcitationSolve**		**ADAPT-VQE**	
	parameter optimization	operator selection	parameter optimization	operator selection
H_2	10^{-6}	10^{-6}	2×10^{-13}	2×10^{-13}
$H_3{}^+$	10^{-6}	10^{-6}	2×10^{-13}	2×10^{-8}
LiH	10^{-7}	10^{-7}	2×10^{-7}	2×10^{-7}
H_2O	10^{-6}	10^{-6}	2×10^{-8}	2×10^{-8}

The experimental setting follows the one of the experiments with the fixed UCCSD ansatz, except that the optimization starts with an empty ansatz. Instead, the fermionic excitation operators from the UCCSD ansatz in its first Trotter-approximation constitute the operator pool for ADAPT-VQE. We employ pool draining, which means that once an operator was selected to extend the ansatz, it is removed from the pool and cannot be used again – thus, the number of ADAPT steps is limited by the size of the pool. We also set a threshold that no more operators are attached when their impact is less than a set threshold value. This value has to be tuned for each molecule individually to achieve optimal convergence. For ExcitationSolve the threshold is an absolute energy difference, for ADAPT-VQE the threshold is a gradient. The chosen values are shown in Table S2.
For ExcSolve2D, the two operators that have the biggest impact are selected, optimized using 2D

ExcitationSolve and then appended to the ansatz. As we already calculated the energies for the individual operators in the ranking, we can reuse those in the optimization. The five single parameter values can be viewed as the shifts of θ_i where $\theta_j = 0$ and vice versa. The value for $\theta_i = \theta_j = 0$ appears in both selections, so only nine values can be recycled. This leaves leaves $25 - 9 = 16$ energy evaluations to be done. Overall we calculate the energies for 24 sets of $(\theta_i,\ \theta_j)$, but the effort is split between operator ranking and actual optimization of the chosen operators.
After each ADAPT step, all parameters are re-optimized until convergence, the corresponding thresholds can again be found in Table S2, they are the same in 1D and 2D ExcitationSolve. ExcitationSolve follows the parameter order in which they were attached. The step sizes for GD in the original ADAPT-VQE counterpart are the same as for the fixed ansatz listed in Table S1.

Supplementary Note 1.4: ExcitationSolve 2D optimization

In the 2D optimization variant of ExcitationSolve, we first perform a sweep over all parameters and rank them based on the difference between their global energy minimum and the Hartree-Fock (HF) energy. This assesses the immediate impact of the operators on improving the HF error. During this initial sweep no parameter values are updated. The top two most impactful parameters are first simultaneously optimized in each VQE iteration, as explained in the "Multi-parameter generalization" subsection in the "ExcitationSolve algorithm" subsection in the Results of the main text, while afterwards all other parameters are optimized independently, i.e., using standard 1D ExcitationSolve. In the evaluation and plotting of the experiments, the initial sweep is included in the total number of energy evaluations, treated equivalently to a VQE iteration in the 1D ExcitationSolve optimization.

Supplementary Note 1.5: Dissociation experiments

We use a fixed UCCSD ansatz in its first Trotter-approximation with fermionic excitations with a Hamiltonian representation in the STO-3G basis set, using all available bond lengths in the Pennylane datasets [5]. The initial state is again the HF state for all configurations. The hyperparameters for the used optimizers are the same as in the "Fixed ansatz (UCCSD) comparison with other optimizers" subsection in the "Experiments" subsection in the Results of the main text. Convergence is defined as approaching the FCI energy up to a certain threshold. As not all molecules converge to the same accuracy, the threshold is specified for each molecule individually depending on the lowest accuracy achieved over all bond lengths (see Table S3).

Table S3: Convergence threshold values for each molecule

	**Threshold**
H_2	5.78×10^{-12} Ha
$H_3{}^+$	2.64×10^{-13} Ha
LiH	2.17×10^{-5} Ha
H_2O	2.27×10^{-3} Ha

Supplementary Note 1.6: Shot noise simulations

The experimental setup is identical to that of the fixed UCCSD ansatz experiments, except that a finite number of shots is used, resulting in energy evaluations being based on estimates rather than exact values. We find that when defining a fixed shot budget, changing the number of shots per energy evaluation or using more energy values for reconstructing the energy landscape has negligible impact on the convergence of ExcitationSolve. Therefore, we use 5 energy values, where we do not reuse the energy for the parameter value $\theta = 0$ but estimate this energy every time by executing the quantum circuit. For COBYLA and BFGS their default termination schemes in the respective `scipy` implementations are used. The optimization of gradient descent and ExcitationSolve was automatically stopped if the energy after the current VQE iteration was larger than the energies after the last two VQE iterations. We use the parameter-shift rule for all gradient-based optimizers.

Supplementary Note 1.7: NISQ hardware experiments

This section describes the IBM-Q implementation specifics and particularly highlights deviations from the fixed and adaptive ansatz experiments in simulation, as previously detailed in Supplementary Notes 1.2 and 1.3, respectively. The execution of quantum circuits on a real NISQ device as opposed to a simulation involves two additional steps, which are the *transpilation* to map the circuit to the hardware specification and constraints, as well as the *error mitigation and suppression* to counteract errors occurring in the execution due to the noisy hardware realization. The specifics are described as follows.

For the transpilation, i.e., the compilation of the quantum circuits from the excitation operator ansatz to the `ibm_quebec` native gate set $\left\{\text{ECR}, I, R_Z(\cdot), \sqrt{X}, X\right\}$ subject to the `ibm_quebec` coupling map, the `qiskit` transpiler service was utilized. The transpilation was configured by setting the `optimization_level` to the maximum level 3 to achieve most optimized circuits at the cost of longer compilation times as part of the pre-processing on the classical computer. Importantly, the transpilation does not only consider the native gate sets and connectivity of the IBM-Q backend into account but optimizes with respect to the gate errors based on the most recent calibration run provided by the `qiskit` runtime service. Especially due to the latter fact, transpilations of the same circuit or ansatz at different times may lead to different outcomes. For all optimizers, close-to-zero parameters are ignored as their expected impact is negligible by removing the associated operator from the circuit before transpilation. This can significantly enhance the energy estimates obtained from the then shallower transpiled circuits. The zero threshold is $1/8$, which sends parameters in the interval $[-1/8, 1/8] \approx [-0.04\pi, 0.04\pi]$, i.e., 4% of the total period of 2π, to zero. Despite the maximum optimization level, the $H_3{}^+$ ansatz was transpiled into exceedingly deep circuits. Therefore, the fermionic double excitations were transpiled manually. We quickly illustrate the technique at hand of a qubit double excitation from orbitals p, q to r, s. We write the generator arising from the JW transformation as

$$G = \frac{1}{8}X_pX_qX_rY_s(I_pI_qI_rI_s + I_pI_qZ_rZ_s - I_pZ_qI_rZ_s - I_pZ_qZ_rI_s - Z_pI_qI_rZ_s - Z_pI_qZ_rI_s + Z_pZ_qI_rI_s + Z_pZ_qZ_rZ_s). \tag{S1}$$

This product decomposition is specifically designed such that the $XXXY$ string commutes with the remaining Z-terms. This would not be the case if one were to factor out, e.g, $XXYY$. Since the Hartree-Fock ground state is represented by only one computational basis state, the Z-terms can be replaced using the eigenvalue relations, therefore reducing the effective generator G to a single Pauli rotation with $G \propto XXXY$. For subsequent excitations, the number of removable strings decreases, until the full 8 strings of G are unavoidable. Thus, the technique is only relevant for the first few excitations. The standard gate decomposition for the single excitations remained. In addition, all Clifford gates in the decomposed circuit were collected and, if at the end of the circuit, absorbed into the Hamiltonian or, otherwise, optimally (heuristically) re-synthesized when acting on at most 3 (more than 3) qubits.

For the error mitigation, the default settings in the `qiskit` runtime for the highest robustness are activated through the currently maximal `resilience_level` of 2. This involves readout error mitigation, Pauli twirling, and zero noise extrapolation. In addition, probabilistic error amplification (PEA) as a more recent and sophisticated noise amplification technique in ZNE [7] is performed with the noise factors $\{1, 1.5, 2\}$. Error suppression is achieved by activating dynamical decoupling, which aims to protect idling qubits through noise-canceling pulse sequences (set to a pulse sequence of two Pauli-X with opposite phases). Idling qubits are particularly common in the transpiled excitation operator circuits due to the high number of two-qubit (ECR) gates with execution times that typically are significantly higher than single-qubit gates. Unless otherwise stated, 8192 shots are spent per energy evaluation.

To limit error propagation, ExcitationSolve is based on five energy evaluations as opposed to of four in simulation to incorporate an energy evaluation of the unshifted parameters instead of re-using the previously determined optimal energy, which match in theory. Since (approximately) non-unique minima can occur in the analytic energy reconstructions, ExcitationSolve is adapted to pick the closer

one. This choice can be justified with the assumption of studying weakly correlated systems at the molecule equilibrium geometries. Although Z-rotations are implemented virtually on IBM devices [8] and thus do not contribute to hardware noise independent of the angle magnitude, selecting the smaller angle reduces circuit depth by skipping the transpilation into additional physical gates in case of parameters close to zero (according to the zero threshold). Furthermore, the coefficients of the two lower frequencies in the analytic energy reconstructions for ExcitationSolve are thresholded at 5×10^{-2} (refit under the corresponding frequency coefficients being set to zero) and if both are set to zero, the period in which the optima are analyzed is halved. The step size in GD is reduced by $1/5$ compared to the ones used in simulation. This more conservative choice showed a decrease in the sensitivity to noisy gradients and the associated risk of divergence in preliminary experiments. To increase the comparability, ExcitationSolve and GD are based on the same parameter equidistant shifts for the analytic energy reconstruction and (four-term) parameter-shift rule, respectively. For the sake of clarity in presenting the experimental results, the number of energy evaluations per parameter for GD is matched with ExcitationSolve to five.

The implementation of the LiH experiments on the IBM-Q device differs from the simulated ones in two size-reducing aspects to compensate for noise further as follows. Hereby, some excitation operators can be removed or at least some spin orbitals (qubits) they act on can be removed, which implicitly decreases the depth of the transpiled circuits. First, certain (occupied) orbitals are *frozen*, which means that these orbitals will be fully occupied. Thus, these orbitals can be excluded from the VQE calculation because they contribute with a constant energy, which can be efficiently computed from the Hamiltonian H classically. For LiH, the lowest two, so-called *core*, spin orbitals are frozen. Second, *qubit tapering* [9, 10] further decreases the qubit requirements below one qubit per spin orbital as per the Jordan-Wigner (JW) mapping [6], which results in six final qubits and ten UCCSD excitation operator LiH. Neither technique is employed for H_2 and $H_3{}^+$ such that the ansatz exactly matches the full UCCSD one of the simulations.

Supplementary Note 2: Experimental results and analyses

The following material provides further details and insight into the performed experiments and analysis of ExcitationSolve.

Supplementary Note 2.1: Hyperparameter tuning results

As found in preliminary experiments, the performance of the gradient-based optimizers is susceptible to a well-tuned step size hyperparameter. We consider the following step sizes: 0.5×10^n and 2.5×10^n for $n \in \{-4, -3, -2, -1, 0\}$. The detailed results for the different runs for the performed hyperparameter tuning as outlined in Supplementary Note 1.1 are presented here in Fig. S1.

Supplementary Note 2.2: ADAPT-VQE resource comparison

Figure S2 displays how well ExcitationSolve and ADAPT-VQE converge to the ground state given a fixed amount of computational resources for the following molecules: H_2, ${H_3}^+$, LiH. On the x-axis is the number of operators that have been attached to the ansatz. As we employ pool-draining, this number is limited by the number of operators in the pool for each molecule. The y-axis shows how often each parameter is re-optimized after each ADAPT-step. Here the threshold is set by a convergence criterion that the change in energy between re-optimizations must be larger than 10^{-6} Ha. An analysis for H_2O has been spared due to exceeding reasonable computational time.

Apart from the overall faster convergence that can be inferred from Fig. 7, ExcitationSolve particularly shines regarding the initialization/warm-start strategy: ExcitationSolve initializes the newly attached operator in its optimal configuration, so it immediately has an impact without any additional energy evaluation overhead. In contrast, the standard ADAPT-VQE initialization sets the parameter to zero, which does not have any direct impact on the energy but heavily relies on the subsequent VQE re-optimization. Indeed, we find that while the GD based algorithm always needs to re-optimize its parameters, further VQE iterations rarely have a substantial impact when using ExcitationSolve. Importantly, this detailed analysis also reveals that ExcitationSolve is capable of finding shorter ansätze, in the case of the more complex molecule LiH, than standard ADAPT-VQE (compare the horizontal transition through the white color, marking chemical accuracy, in the bottom two plots of Fig. S2 in a region where the VQE re-optimization is converged.) This hints towards the advantage of the globally-informed operator selection criterion with ExcitationSolve as opposed to the local gradient-based criterion in the original ADAPT-VQE.

Supplementary Note 2.3: ADAPT-VQE impact analysis of operator selection criterion vs parameter optimizer

To separate the respective impacts of the optimizers and the operator selection criteria on the convergence, in Fig. S3 we compare adaptive ansatz optimizations with combinations of GD as parameter optimizer and ExcitationSolve as operator selector, and, vice versa, with pure gradient- and ExcitationSolve-based ADAPT-VQE. It is apparent that while using ExcitationSolve for both the optimization and selection is still the most efficient method. Already replacing only one part with ExcitationSolve leads to a great speed-up over the original (purely gradient-based) ADAPT-VQE. The reason that convergence using GD is much faster when using ExcitationSolve as operator selector already is its initialization strategy. Initializing the newly selected operator with a parameter $\theta_i \neq 0$ that is often near-optimal with respect to all parameters $\boldsymbol{\theta}$ naturally reduces the number of GD steps required to reach convergence. This is in excellent agreement with the observation in Fig. S2, which shows that little re-optimization is required when using ExcitationSolve for the selection and initialization.

For LiH in Fig. S3a, one can clearly identify a region (shortly after chemical accuracy was reached) in which the gradient-based selection criterion overestimates the number of operators required to reach a certain energy threshold – independent of the optimization strategy. This results in slower convergence in the start. However, once this “plateau” is breached, using ExcitationSolve as the optimizer provides a significant advantage over GD.

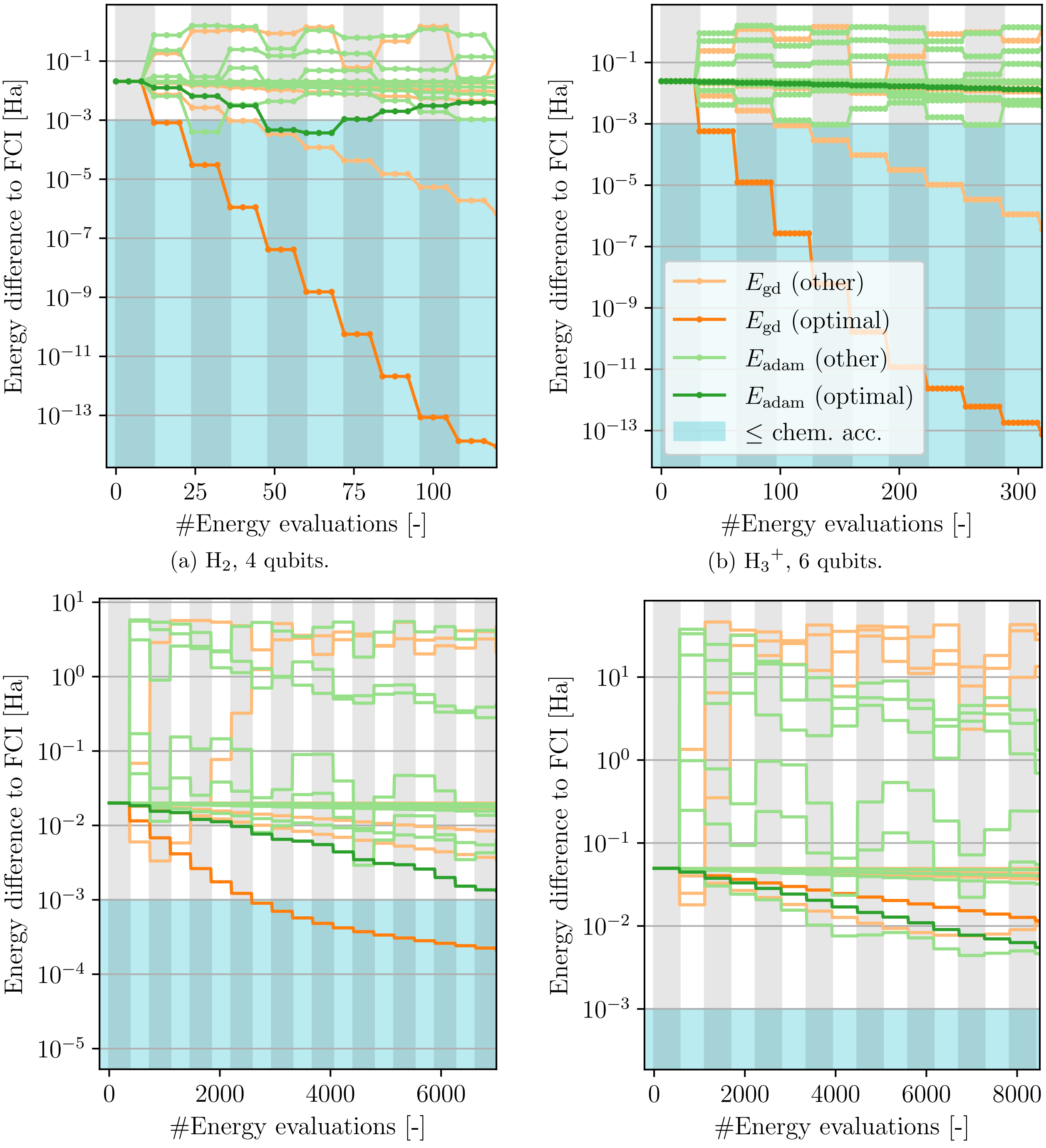

(a) H_2, 4 qubits.

(b) $H_3{}^+$, 6 qubits.

(c) LiH, 12 qubits.

(d) H_2O, 14 qubits.

Figure S1: **Comparison of optimizer step sizes.** The optimizers under consideration are Gradient descent (yellow) and Adam (green). The plots show the error of the VQE with respect to the FCI solution $|E_{VQE} - E_{FCI}|$ over the number of energy evaluations for all tested step sizes. The optimal step size is highlighted. The light blue region signifies the chemical accuracy (10^{-3} Ha) and the alternating vertical shading marks each iteration over all parameters.

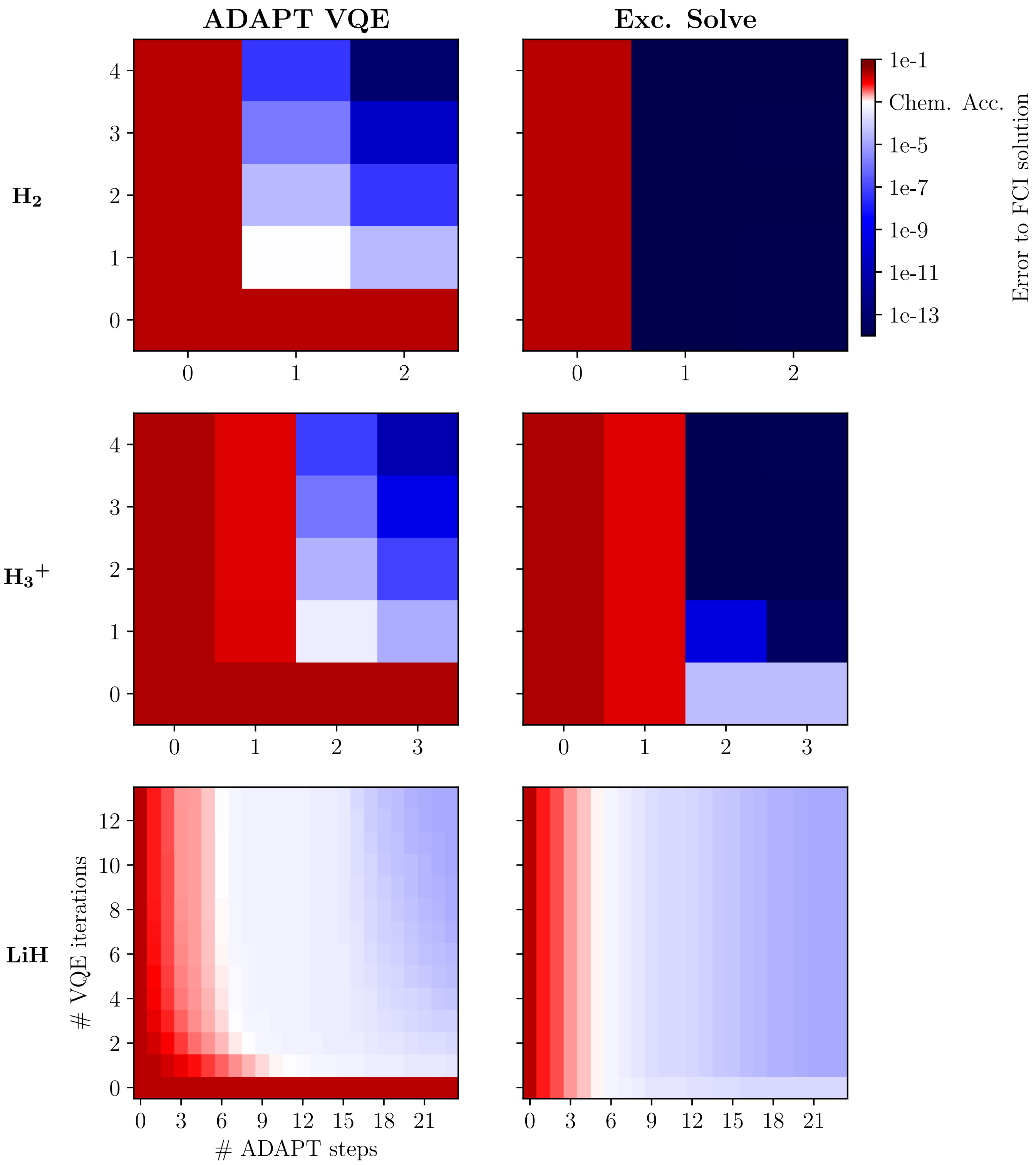

Figure S2: **Evaluation of ExcitationSolve for ADAPT-VQE.** ExcitationSolve (right) in an adaptive setting compared with the original ADAPT-VQE (left) based on GD for molecules (top to bottom) H_2, H_3^+, LiH. On both axes are the resources spent on the calculation: The number of ADAPT steps signals how many operators have been appended to the ansatz, the number of VQE iterations indicates how often each of the parameters has been optimized in each ADAPT step. The color code signals how close the result is to the exact FCI solution.

In both cases of LiH in Fig. S3a and H_2O in Fig. S3b, in comparison with the original ADAPT-VQE, we find that using gradient-selection along with ExcitationSolve optimization yields faster convergence but the opposite approach leads to an improved operator selection quality and, hence, fewer operators in the ansatz (cf. Table S4). In any case, it is therefore advisable to employ ExcitationSolve for both methods to profit from both the convergence speed-up and the reduction in circuit depth.

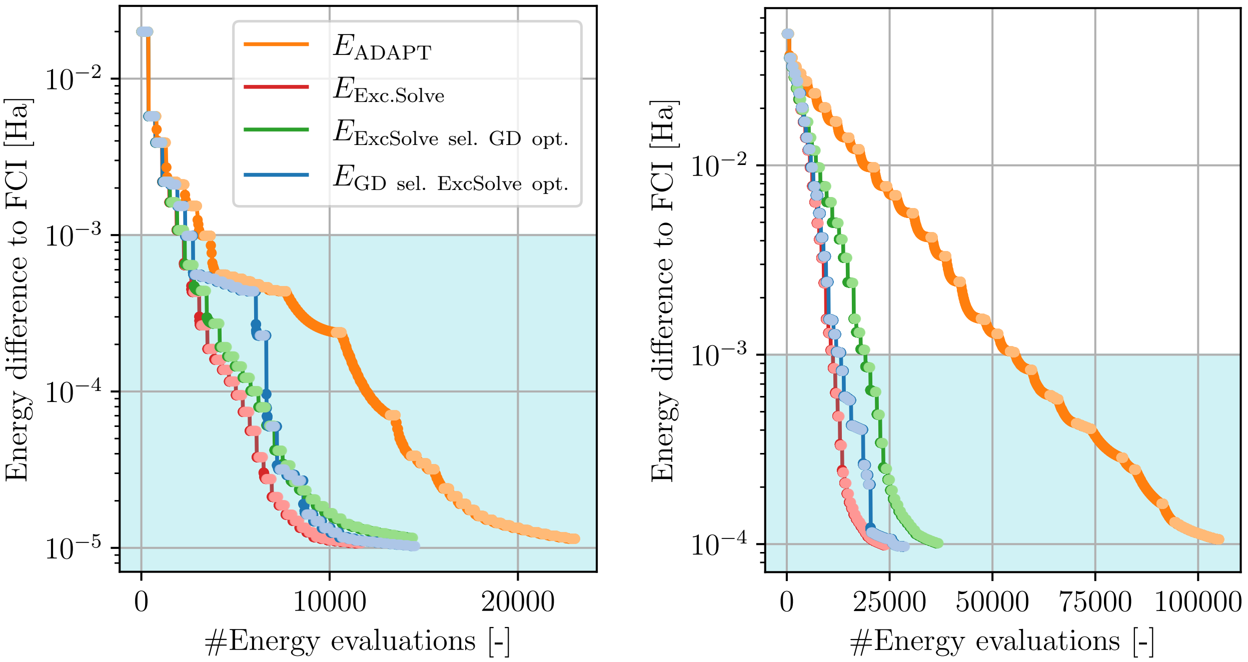

(a) LiH, 12 qubits.

(b) H_2O, 14 qubits.

Figure S3: **Comparison of the impact of optimizer and selection method for adaptive ansätze.** In addition to the standard ExcitationSolve and ADAPT-VQE optimization, one setting uses ExcitationSolve only for the operator selection but GD for the parameter optimization (green), and, vice versa, the original (gradient-based) ADAPT-VQE operator selection and ExcitationSolve for the parameter optimization in another setting (blue). Experiments are for the molecules a) LiH and b) H_2O. The light blue region signifies the chemical accuracy (10^{-3} Ha).

Table S4: **Number of operators selected for all four combinations in adaptive optimization impact study for LiH and H_2O.** Combinations are labeled selector/optimizer. Indicates numbers of operators to reach full convergence and, in parentheses, chemical accuracy.

Molecule	ExcSolve/ExcSolve	ExcSolve/GD	GD/ExcSolve	GD/GD
LiH	30 (6)	31 (6)	34 (6)	34 (6)
H2O	42 (20)	42 (20)	48 (22)	48 (22)

Supplementary Note 2.4: Dissociation curve energy errors

Here we present the final energy differences to FCI when convergence is reached for our dissociation curves from the "Dissociation curves" subsection in the "Experiments" subsection in the Results of the main text. The energy differences are the same for all optimizers but depend on the molecule. The final energy differences for each molecule and bond distance are shown in Figure S4. For H_2 and $H_3{}^+$ the final energy differences are below 10^{-11} Ha and 10^{-12} Ha, respectively. For LiH and H_2O the final energy differences vary from about 5×10^{-6} Ha to about 3×10^{-5} Ha and from about 1×10^{-5} Ha to about 3×10^{-3} Ha based on the bond distance, respectively. For the bond distances 2.02 Å and

2.06 Å in H_2O we ignored the cases where ExcitationSolve got stuck in local minima when computing the final energy differences. These two cases are separately discussed in Supplementary Note 2.5.

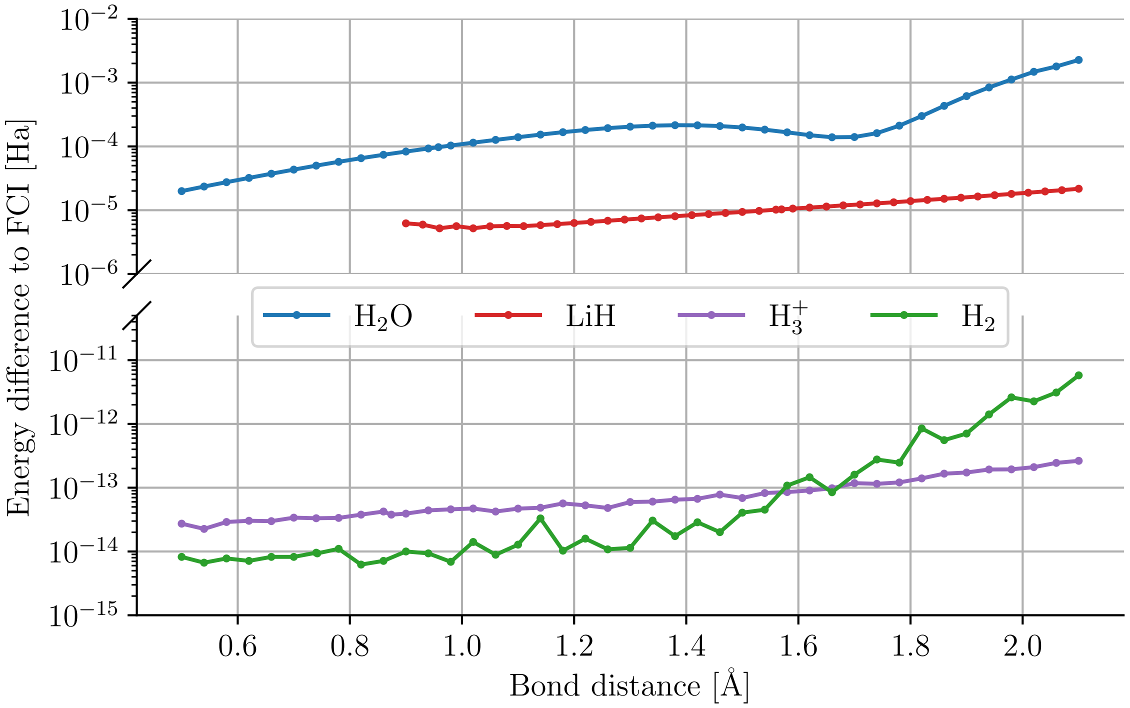

Figure S4: Final energy differences to FCI when convergence is reached for each molecule and bond distance shown in Figure 8.

Supplementary Note 2.5: Analysis of dissociation curve experiments on local minima avoidance

Particular attention should be drawn to the two data points of the H_2O dissociation curve (Fig. 8d) at the bond distances of 2.02 Å and 2.06 Å. The convergence analysis for these data points can be inferred from Fig. S5. As detailed in Supplementary Note 1.2, all parameters have been optimized in their order of appearance in the UCCSD ansatz, i.e., a fixed order. Figures S5a and S5b depict cases in which this order causes ExcitationSolve to get stuck either in local minima or extremely flat parts of the optimization landscape, thus failing to converge within a reasonable number of energy evaluations. Meanwhile, Fig. S5a also provides further evidence for the utility of 2D optimization, which successfully converges to the global minimum within the expressivity of the ansatz. While the 2D optimization can be utilized to avoid local minima, our heuristic to simultaneously optimize the two most impacting parameters may not succeed in some rare instances. We have found one example in Fig. S5b, where both the 1D and 2D optimizers get stuck. Fortunately, randomly shuffling the parameter order in each VQE iteration while performing solely 1D optimization achieves convergence for both cases. Randomly shuffling the parameter order means that we randomly change the order in which we optimize the parameters at the beginning of each VQE iteration, while all parameters are optimized using ExcitationSolve 1D optimization. We note that this shuffling does not guarantee convergence to the optimal parameters in general and here we show only one instance of shuffling where it succeeded. We did not find a systematic way of deciding when to apply shuffling to avoid local minima and leave this for future work. In the case where the 2D optimizer already converges (Fig. S5a), it is still notably faster than the shuffled 1D approach.

Supplementary Note 2.6: Shot noise

We repeat the experiments from the "Fixed ansatz (UCCSD) comparison with other optimizers" subsection in the the "Experiments" subsection in the Results of the main text with shot noise instead

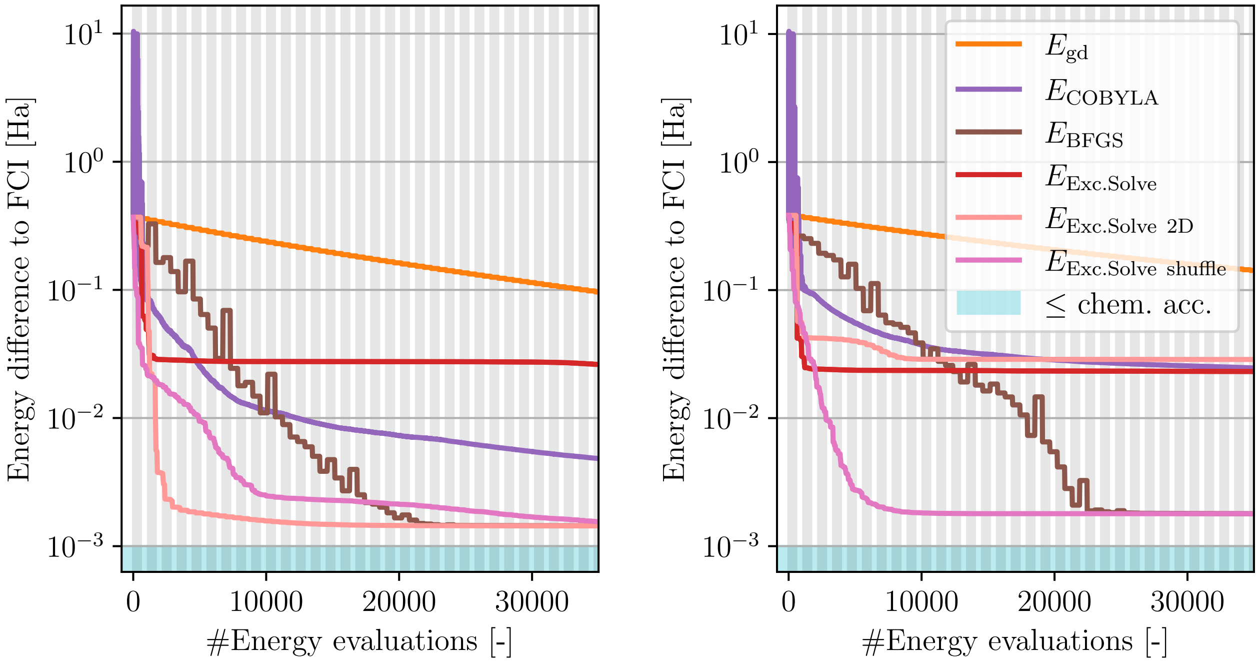

(a) H_2O, 14 qubits, bond distance 2.02 Å.

(b) H_2O, 14 qubits, bond distance 2.06 Å.

Figure S5: **Optimization of H_2O for two specific bond distances.** The optimizers under consideration are ExcitationSolve (red), COBYLA (purple), Gradient descent (yellow) and BFGS (brown). The plots show the error of the VQE with respect to the FCI solution $|E_{\mathrm{VQE}} - E_{\mathrm{FCI}}|$ over the number of energy evaluations for the bond distances marked separately in Fig. 8d where parameter shuffling is used. The light blue region signifies the chemical accuracy (10^{-3} Ha) and the alternating vertical shading marks each iteration over all parameters.

of exact state vector simulations. Due to the large number of shots needed to achieve chemical accuracy and the increasing computation time for larger molecules, we restrict ourselves to the molecules H_2 and ${H_3}^+$. We perform 10^7 shots for all optimizers and molecules in the results we show here since the overall qualitative behavior of the optimizers was rather independent of the number of shots. Implementation details can be found in Supplementary Note 1.6. Note that, unlike in the results presented in the main text, the parameter order considered here comprises of first the single then the double excitations in the UCCSD ansatz, which is slightly sub-optimal in the first iteration as the single excitations cannot cause any change and results in a shifted energy reduction pattern in the plots.

Figure S6 presents the results. For optimizers that do not update parameters at each energy evaluation, we repeatedly plot the latest updated energy which results in energy plateaus which seem not affected by noise. For example, GD has these plateaus are during the calculation of the gradient. We find that the shot noise has a significant impact on all used optimizers and limits the achievable accuracy. The difference between the optimizers is less pronounced than in the state vector simulations form the "Fixed ansatz (UCCSD) comparison with other optimizers" subsection in the "Experiments" subsection in the Results of the main text. Overall, the results are similar to the state vector simulations, only that the maximum achievable accuracy of every optimizer is limited by the shot noise. We see that ExcitationSolve reaches its maximum accuracy within similar number of energy evaluation as in the state vector simulations. This includes H_2, where ExcitationSolve achieves its maximum accuracy within one VQE iteration. Most importantly, ExcitationSolve reaches its maximum accuracy faster than all other optimizers. With this, we note that the convergence speed of ExcitationSolve is robust against noise.

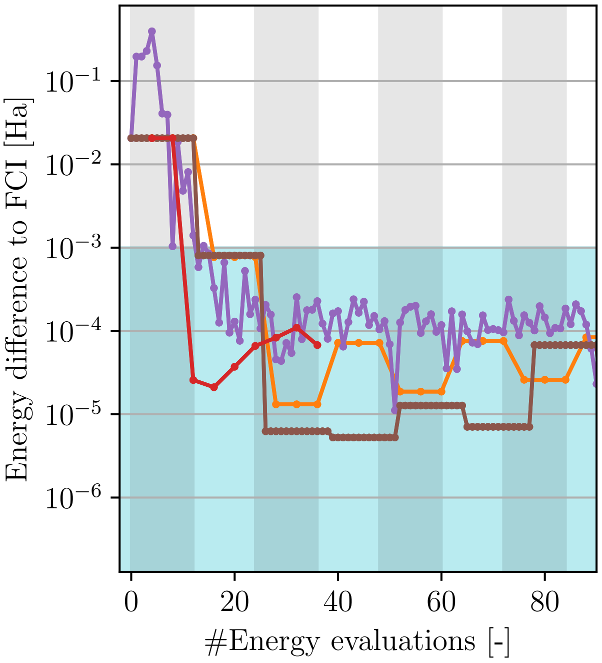

(a) H_2, 4 qubits.

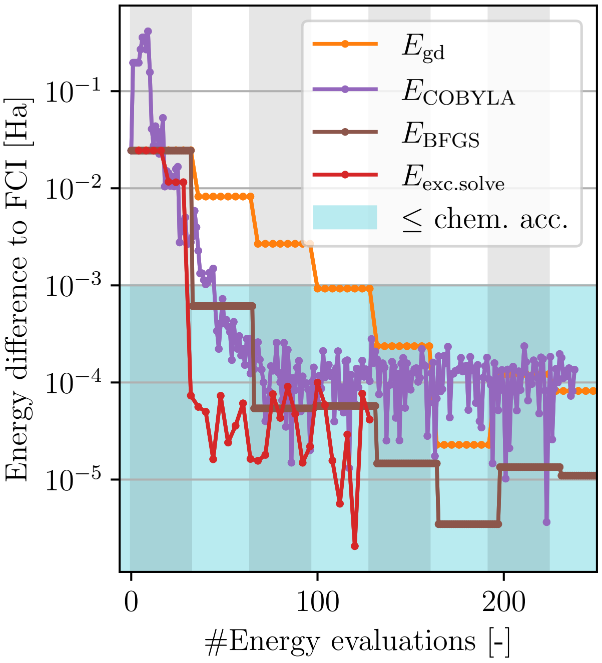

(b) $H_3{}^+$, 6 qubits.

Figure S6: **Comparison of optimizers under the influence of shot noise.** The optimizers under consideration are ExcitationSolve (red), COBYLA (purple), Gradient descent (yellow) and BFGS (brown) with 10^7 shots each. The plots show the error of the VQE with respect to the FCI solution $|E_{\mathrm{VQE}} - E_{\mathrm{FCI}}|$ over the number of energy evaluations for the molecules H_2 (Fig. 6a), $H_3{}^+$ (Fig. 6b). The light blue background signifies when chemical accuracy has been reached. Vertical lines mark when one iteration over all parameters has been completed.

Supplementary Note 2.7: NISQ robustness of ExcitationSolve adaptive operator ranking

To study the NISQ robustness of the ExcitationSolve operator ranking for adaptive ansatz optimization compared to ADAPT-VQE, we analyze the permutations from the true operator rankings via slope charts in Fig. S7, which connect matching operators in different rankings. There, it becomes apparent that the operator ranking is reproduced more accurately on the IBM-Q device via the ExcitationSolve than the gradient-based ADAPT-VQE scores. Since the rankings produced by both methods match under exact simulation, this implies that the ExcitationSolve scores are more robust against noise in reproducing the true ranking. Operators with zero ExcitationSolve scores cannot promote an energy decrease and should not be selected. However, ADAPT-VQE frequently mixes such operators with contributing (non-zero score) operators in the ranking from IBM-Q evaluations. In the ExcitationSolve ranking, such a confusion only occurs once and otherwise provides a clear separation between contributing and non-contributing operators. Furthermore, ExcitationSolve picks an operator ranked higher in simulation as the top choice than ADAPT-VQE and puts the exact top operator second instead of third. Overall, ExcitationSolve seems more likely to append operators that can contribute with a higher energy decrease than the original ADAPT-VQE when evaluated on noisy hardware.

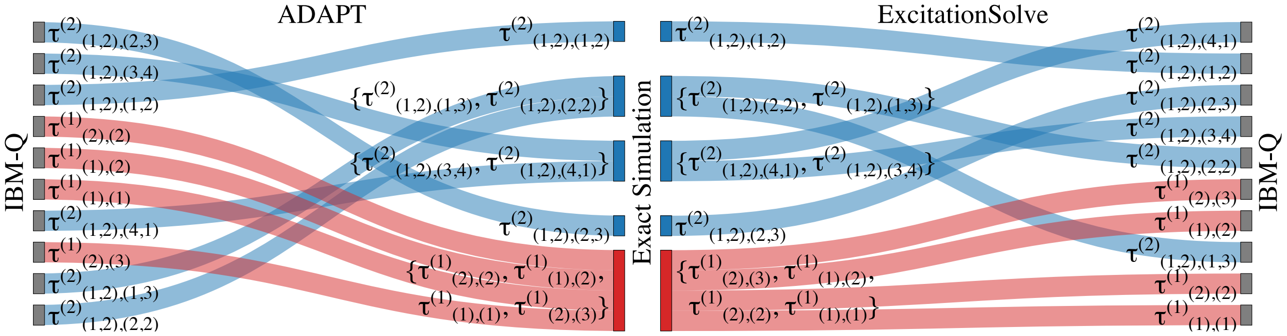

Figure S7: **Benchmarks on NISQ (`ibm_quebec`) quantum processor for adaptive ansätze.** The excitation operator rankings for adaptive ansätze on the initial HF states of LiH (frozen core, tapered) calculated on the `ibm_quebec` device are visualized. The operator scores and resulting noisy rankings for ExcitationSolve (right) and ADAPT-VQE (left) are compared by visualizing their permutations with the true rankings in the middle. The true scores are exactly simulated, where red (blue) indicates zero (non-zero) scores.

Supplementary Note 3: ExcitationSolve algorithmic details

Algorithmic details for ExcitationSolve for the application to both fixed and adaptive ansätze are provided in the following in form of pseudo code. For implementations of ExcitationsSolve and its variants/extensions in `Python`, refer to the code availability statement in the main text. We also include additional extensions and methodologies for ExcitationSolve in this Supplementary Note that are not present in the experimental studies.

Supplementary Note 3.1: ExcitationSolve for fixed ansätze

Algorithm 2 outlines the ExcitationSolve optimization algorithm for fixed ansätze. Here, the k iterations reflect the number of parameter updates that have been performed by ExcitationSolve. Hence, the parameters of a new iteration k are initialized by the parameters of the previous iteration $k-1$, i.e., $\boldsymbol{\theta}^{(k)} \leftarrow \boldsymbol{\theta}^{(k-1)}$. Importantly, only the line highlighted in purple requires quantum hardware (QC), while everything else is computed efficiently on a classical device. Note that the energy associated with the unshifted current parameter value $\theta_j^{(k)}$ is re-used from the previous iteration or, in the first iteration, from the initial HF energy (CC). The order in which the N parameters are iterated over in the *for-each* loop can be chosen freely. To clearly indicate that the parameter indices and the sweep order are independent, we use two distinct iterators, j and k.

Algorithm 1: ExcitationSolve optimization algorithm for fixed ansätze.

Hardware Resources: Quantum Computer (QC), Classical Computer (CC)

Input: Initial parameters $\boldsymbol{\theta}^{(0)} = \mathbf{0}$, HF/init. energy $E^{(0)} = f(\boldsymbol{\theta}^{(0)})$, HF/init. state $|\psi_0\rangle$, fixed ansatz $U(\cdot)$ *(excitation operators defined as in the Results of the main text)*

Output: Optimized parameters $\boldsymbol{\theta}^*$ and energy E^*

$k = 0$;
repeat
  foreach Parameter θ_j **do**
    $k \leftarrow k + 1$;
    Keep other parameters $\theta_{l \neq j}^{(k)}$ fixed;
    Re-use optimal energy from previous iteration $k-1$ as energy evaluation in current iteration $E_0^{(k)} = E^{(k-1)}$ for un-shifted parameter position $\theta_{j,0}^{(k)} = \theta_j^{(k)}$;
    Determine energies $E_1^{(k)}, E_2^{(k)}, E_3^{(k)}, E_4^{(k)}$ at four additional parameter positions $\theta_{j,1}^{(k)}, \theta_{j,2}^{(k)}, \theta_{j,3}^{(k)}, \theta_{j,4}^{(k)}$, e.g., equidistant positions $\theta_{j,l}^{(k)} = \theta_j^{(k)} + 2\pi l/5$ for $l = 1, \ldots, 4$ *(see Eq. (1) via QC)*;
    Reconstruct energy landscape in parameter θ_j by solving linear equation system of five $(l = 0, \ldots, 4)$ equations $f_{\boldsymbol{\theta}^{(k)}}(\theta_{j,l}^{(k)}) \overset{!}{=} E_l^{(k)}$ *(see Eq. (3))*;
    Determine global minimum of reconstruction $E^{(k)} = \min_{\theta_j} f_{\boldsymbol{\theta}^{(k)}}(\theta_j)$ and update parameter $\theta_j^{(k)} \leftarrow \arg\min_{\theta_j} f_{\boldsymbol{\theta}^{(k)}}(\theta_j)$ *(see companion matrix method in the Methods)*;
  end
until Convergence (threshold energy reduction $|E^{(k-N)} - E^{(k)}| \leq \epsilon$) $\rightarrow \boldsymbol{\theta}^* = \boldsymbol{\theta}^{(k)}, E^* = E^{(k)}$;

Supplementary Note 3.2: ExcitationSolve for ADAPT-VQE (adaptive ansätze)

Algorithm 2 details the application of ExcitationSolve to ADAPT-VQE (adaptive ansätze). Here, ADAPT iteration ℓ denotes how many operators have been appended to the ansatz, while the number of update steps for re-optimizing all parameters in-between ADAPT iterations is omitted by calling ExcitationSolve for a fixed ansatz (Algorithm 1). The index m describes the index of the operators in the operator pool $\mathcal{P}$. Importantly, the usage of the quantum device solely happens in Algorithm 1 when invoked as sub-routines.

Algorithm 2: ExcitationSolve for ADAPT-VQE (adaptive ansätze).

Hardware Resources: Quantum Computer (QC), Classical Computer (CC)

Input: Initial (empty) parameters $\boldsymbol{\theta}^{(0)} = \varnothing$, Empty ansatz $U^{(0)}(\cdot) = I$, HF/init. energy $E^{(0)} = f(\boldsymbol{\theta}^{(0)})$, HF/init. state $|\psi_0\rangle$, Pool of excitation operators $\mathcal{P}$ *(excitation operators defined as in the Results of the main text)*

Output: Optimized parameters $\boldsymbol{\theta}^*$ and energy E^*

$\ell = 0$;
while *True* **do**
  $\ell \leftarrow \ell + 1$;
  foreach Operator in pool $U_m(\cdot) \in \mathcal{P}$ **do**
    New candidate ansatz by appending operator $U^{(\ell-1)}(\boldsymbol{\theta}^{(\ell-1)}) \circ U_m(\theta_m)$;
    Evaluate operator candidate via minimum energy and optimal parameter $E_m^{(\ell)}, \theta_m^{(\ell)} \leftarrow$ inner loop in Algorithm 1 incl. QC (fix previous parameters $\boldsymbol{\theta}^{(\ell-1)}$);
  end
  if Convergence (threshold energy reduction $|E^{(\ell-1)} - \min_m E_m^{(\ell)}| \leq \epsilon$) **then**
    $\boldsymbol{\theta}^* = \boldsymbol{\theta}^{(\ell-1)}, E^* = E^{(\ell-1)}$;
    break;
  end
  Select operator with strongest energy reduction $m^* = \arg\min_m E_m^{(\ell)}$ to extend ansatz $U^{(\ell)}(\boldsymbol{\theta}^{(\ell)}) = U^{(\ell-1)}(\boldsymbol{\theta}^{(\ell-1)}) \circ U_m^*(\theta_{m^*}^{(\ell)})$ and optimally initialize the new parameter $\boldsymbol{\theta}^{(\ell)} = \boldsymbol{\theta}^{(\ell-1)} \cup (\theta_{m^*}^{(\ell)})$;
  Re-optimize all parameters via ExcitationSolve under fixed ansatz $E^{(\ell)}, \boldsymbol{\theta}^{(\ell)} \leftarrow$ Algorithm 1 incl. QC;
end

Supplementary Note 3.3: ExcitationSolve for multiple occurrences of multiple parameters

After having explored the two cases of multiple distinct parameters and multiple occurrences of a single parameter in the main text, it remains to explore the most general case: multiple occurrences of multiple parameters (different parameters may appear different number of times). The result is a straightforward conclusion of both previous results. Each unique parameter θ_i introduces one dimension and the order of the Fourier series in the corresponding dimension is given by the respective number of occurrences. Let $\tilde{\boldsymbol{\theta}} \subseteq \boldsymbol{\theta}$ be the subset of simultaneously varied parameters and S_i be the number of occurrences of a parameter $\theta_i \in \tilde{\boldsymbol{\theta}}$. The energy landscape can then be expressed as

$$f_{\boldsymbol{\theta}}(\boldsymbol{\theta}_{\tilde{\theta}}) = \boldsymbol{c} \cdot \left[\bigotimes_{\theta_i \in \tilde{\boldsymbol{\theta}}} (\cos(\theta_i), \cos(2\theta_i), \ldots, \cos(2S_i\theta_i), \sin(\theta_i), \sin(2\theta_i), \ldots, \sin(2S_i\theta_i), 1)^\top \right], \qquad \text{(S2)}$$

where $\boldsymbol{c}$ is a real-valued vector with dimension $\prod_{\theta_i \in \tilde{\boldsymbol{\theta}}} (4S_i + 1)$.

Supplementary Note 3.4: Reconstruction strategies for noise robustness

As the analytic energy landscape is resembled by a second-order Fourier series as in Eq. (3), five energy evaluations set the minimum requirement to uniquely determine the five coefficients. However, the energy landscape reconstruction in ExcitationSolve can be readily extended beyond five energy evaluations. Assuming that the energy evaluations are inexact (e.g. due to device- or shot-noise), this approach can make ExcitationSolve more robust against noise.

The then overdetermined linear equation system can then be solved in two ways: Using the least-squares method or a discrete Fourier transform. From a statistical perspective, the least-squares method is solving the regression problem in a second-order Fourier basis expansion [11]. Then, in terms of maximum-likelihood optimality, the least-squares estimation yields the optimal result under a normally distributed noise assumption [12]. This assumption is approximately fulfilled for pure shot-noise with practical shot numbers [13], yet only sometimes observed for hardware noise [14]. The discrete Fourier transform truncated at the second order could be utilized because higher frequencies cannot be contained in the energy landscape as in Eq. (3) but are solely subject to noise. Both approaches can in fact be seen as equivalent if the parameter-shifts are equidistant, which is necessary for the Fourier transform and generally suggested [15]. The equivalence can be supported by the least-squares guarantee when solving the regression problem [12] and the *best approximation* principle of the truncated Fourier transform [16]. Both arguments are made under the L^2 norm and exhibit the geometrical interpretation of orthogonal projections on the feasible function space [16, 17].

From a practical perspective, the possibility of using more than five energy values raises the following question: Given a fixed shot budget T, should we rather spend more shots per energy evaluation or query more energy values for the best energy reconstruction? The corresponding variance of the energy estimate when allocating t shots per evaluation for any parameter position $\theta_j \in [-\pi, \pi]$ is given by $(\Delta H)^2/t$ [13] where (one-shot) observable variance $(\Delta H)^2$ depends on the prepared quantum state and, consequently, on the choice of parameters $\boldsymbol{\theta}$. To simplify the subsequent discussion, we assume $(\Delta H)^2$ to be constant and incorporate it into a proportionality constant. For the total shot budget T, we perform $t = T/K$ shots for each of the K energy evaluations[1]. Then, each energy evaluation is estimated with a noise variance of $\sigma^2 \sim \frac{1}{T/K}$, which leads to an average variance of these K estimates of $\sigma_T^2 \sim \sigma^2/K$. Therefore, this average estimate variance becomes $\sigma_T^2 \sim 1/T$, i.e., inverse-proportional to the total shot budget T and, importantly, independent of K. In conclusion, the distribution of shots per energy evaluation under a fixed shot budget will not quantitatively change the information extracted from the quantum computer and, thus, does not impact the quality of the reconstruction. On the other hand, if we relax the assumption of a constant observable variance $(\Delta H)^2$, which is certainly expected in practice, we cannot make general statements about a trade-off between the shot count and number of energy evaluations. Otherwise, $(\Delta H)^2$ must be known (again of a finite Fourier series form), however, its estimation could pose a significant challenge in practice due to the likely high number of terms in H^2. It must be emphasized that our analysis is intentionally kept simple and focuses solely on the average statistical error of the energy evaluation estimates, which serve as the basis for the reconstruction. A detailed analysis of how the error propagates through this reconstruction to parameter regions away from the sampling points, and particularly its dependence on the number and spacing of these points for the reconstruction, is beyond the scope of this work. For a more rigorous treatment, we refer the reader to Ref. [15], which, for example, identifies three equiangular (i.e., equally spaced) sampling points on the unit circle as an optimal configuration for Rotosolve.

[1] For integer devision, assume compatible $T, K, t \in \mathbb{N}_{>0}$.

Supplementary Note 4: Proofs

Supplementary Note 4.1: Analytic energy function in single parameter

We present three proofs that show that the energy function in Eq. (1) has the analytical form of a second-order Fourier series as in Eq. (3). First, a constructive proofs is presented, which also shows the explicit connection of the coefficients a_1, a_2, b_1, b_1, c in Eq. (3) and (expectation values of) observables of variationally prepared states.

Proof. The excitation operators $U(\theta)$ as defined in Eq. (2) have a Hermitian generator G with the property $G^3 = G$. The exponential series simplifies to the Euler formula

$$U(\theta) = \exp(-i\theta G) = I + (\cos(\theta) - 1)\, G^2 - i\sin(\theta) G. \tag{S3}$$

because $G^3 = G$ [18]. This property can also be motivated through a hidden SU(2) symmetry associated with operators of the type $G^3 = G$, as already studied for excitation operators in Refs. [19–22]. When varying the parameter θ_j of a single excitation operator, while leaving all the other parameters $\theta_{i<j}$ and $\theta_{i>j}$ of preceding and succeeding excitation operators, respectively, in the circuit $U(\boldsymbol{\theta}) = \prod_k U(\theta_k)$ fixed. Hence, these operators can be subsumed in the input state

$$\left(\prod_{i<j} U(\theta_i)\right) |\psi_0\rangle =: |\psi'\rangle \tag{S4}$$

and observable

$$\left(\prod_{i>j} U(\theta_i)\right)^\dagger H \left(\prod_{i>j} U(\theta_i)\right) =: H', \tag{S5}$$

respectively, allowing us to re-phrase the energy function of Eq. (1) through the following expectation value

$$f_{\boldsymbol{\theta}}(\theta) = \langle \psi' | U^\dagger(\theta) H' U(\theta) | \psi' \rangle\,. \tag{S6}$$

The dependence of the state $|\psi'\rangle$ and observable H' on the remaining parameters is omitted, as well as the index of the varied parameter $\theta_j = \theta$, for the sake of clarity. Inserting now the Euler formula in Eq. (S3) into Eq. (S6) yields

$$f_{\boldsymbol{\theta}}(\theta) = \left\langle \left(I + (\cos(\theta) - 1)\, G^2 + i\sin(\theta) G\right) H' \left(I + (\cos(\theta) - 1)\, G^2 - i\sin(\theta) G\right) \right\rangle \tag{S7}$$

$$\begin{aligned} = \quad & \left\langle \left\{H', G^2\right\} \right\rangle (\cos(\theta) - 1) + \left\langle G^2 H' G^2 \right\rangle (\cos(\theta) - 1)^2 \\ & + \left\langle i\left[GH'G, G\right] \right\rangle (\cos(\theta) - 1)\sin(\theta) \\ & + \left\langle i\left[G, H'\right] \right\rangle \sin(\theta) + \left\langle GH'G \right\rangle \sin^2(\theta) \\ & + \left\langle H' \right\rangle \end{aligned} \tag{S8}$$

$$\begin{aligned} = \quad & \left(\left\langle \left\{H', G^2\right\} \right\rangle - 2\left\langle G^2 H' G^2 \right\rangle\right) \cos(\theta) \\ & + \left\langle i\left[GH'G, G\right] \right\rangle \sin(\theta)\cos(\theta) \\ & + \left(\left\langle i\left[G, H'\right] \right\rangle - \left\langle i\left[GH'G, G\right] \right\rangle\right) \sin(\theta) \\ & + \left\langle G^2 H' G^2 \right\rangle \cos^2(\theta) \\ & + \left\langle GH'G \right\rangle \sin^2(\theta) \\ & + \left\langle H' \right\rangle - \left\langle \left\{H', G^2\right\} \right\rangle + \left\langle G^2 H' G^2 \right\rangle, \end{aligned} \tag{S9}$$

where all expectation values above are to be understood with respect to $|\psi'\rangle$, i.e., $\langle \cdot \rangle = \langle \psi' | \cdot | \psi' \rangle$. Considering the three trigonometric identities, *Pythagorean trigonometric identity* $\cos^2(\theta) + \sin^2(\theta) = 1$, *double-angle-formula* $\sin(\theta)\cos(\theta) = \sin(2\theta)/2$, and *power-reduction-formula* $\sin^2(\theta) = (1 - \cos(2\theta))/2$,

we obtain

$$\begin{aligned} f_{\boldsymbol{\theta}}(\theta) = \quad & \underbrace{\left(\left\langle\left\{H', G^2\right\}\right\rangle - 2\left\langle G^2 H' G^2\right\rangle\right)}_{=a_1}\cos(\theta) \\ & + \underbrace{\tfrac{1}{2}\left(\left\langle G^2 H' G^2\right\rangle - \left\langle G H' G\right\rangle\right)}_{=a_2}\cos(2\theta) \\ & + \underbrace{\left(\left\langle i\left[G, H'\right]\right\rangle - \left\langle i\left[G H' G, G\right]\right\rangle\right)}_{=b_1}\sin(\theta) \\ & + \underbrace{\tfrac{1}{2}\left\langle i\left[G H' G, G\right]\right\rangle}_{=b_2}\sin(2\theta) \\ & + \underbrace{\left\langle H'\right\rangle - \left\langle\left\{H', G^2\right\}\right\rangle + \tfrac{1}{2}\left(\left\langle G H' G\right\rangle + 3\left\langle G^2 H' G^2\right\rangle\right)}_{=c}. \end{aligned} \tag{S10}$$

Recognizing that Eq. (S10) precisely matches the form of a second-order Fourier series for the energy function in a single parameter as in Eq. (3) concludes the proof. □

Second, we provide an alternative proof of this connection between Eq. (1) and Eq. (3), given the theory of general parameter-shift rules [23], which links the eigenvalues of G to the frequencies present in the energy function.

Proof (alternative I). Given the eigenvalues $\{\omega_i\}$ of the Hermitian generator G of a single parameterized operator $U(\theta) = \exp(i\theta G)$, Ref. [23] determines that the energy function Eq. (1) can be written in the form of a finite Fourier series

$$f_{\boldsymbol{\theta}}(\theta_j) = a_0 + \sum_{\ell=1}^{R} a_\ell \cos\left(\Omega_\ell \theta_j\right) + \sum_{\ell=1}^{R} b_\ell \sin\left(\Omega_\ell \theta_j\right) \qquad \text{(Ref. [23], Eq. (6))}$$

of the order $\max_\ell\{\Omega_\ell\}$. Here, they introduce the R *unique positive differences* $\{\Omega_\ell\} := \{\omega_k - \omega_h \mid \omega_k > \omega_h\}$. In the case of the excitation operators, we exploit the fact that a Hermitian generator with the property $G^3 = G$ must have eigenvalues $\omega_k \in \{-1, 0, 1\}$ [18]. If all three different possible eigenvalues are contained in the spectrum, $R = 2$ unique positive differences $\{\Omega_\ell\} = \{1, 2\}$ are present, which proves that Eq. (1) is a second-order Fourier series Eq. (3) when varied in a single parameter θ_j. We now prove that the spectrum of the Hermitian generators of excitation operators $G = i\tau^{(m)}_{\boldsymbol{o},\boldsymbol{v}}$ does indeed contain all three possible eigenvalues $\{-1, 0, 1\}$. For the eigenvalues $\omega = \pm 1$, we may construct the corresponding eigenstates explicitly as

$$|\pm\rangle = \frac{1}{\sqrt{2}}\left(|0_{\boldsymbol{v}_1} 0_{\boldsymbol{v}_2} \ldots 0_{\boldsymbol{v}_m} 1_{\boldsymbol{o}_1} 1_{\boldsymbol{o}_2} \ldots 1_{\boldsymbol{o}_m}\rangle \pm i\,|1_{\boldsymbol{v}_1} 1_{\boldsymbol{v}_2} \ldots 1_{\boldsymbol{v}_m} 0_{\boldsymbol{o}_1} 0_{\boldsymbol{o}_2} \ldots 0_{\boldsymbol{o}_m}\rangle\right). \tag{S11}$$

Then, we have

$$\begin{aligned} G\,|\pm\rangle = i\tau^{(m)}_{\boldsymbol{o},\boldsymbol{v}}\,|\pm\rangle &= i\left(a^\dagger_{v_1} a^\dagger_{v_2} \ldots a^\dagger_{v_m}\; a_{o_m} \ldots a_{o_2} a_{o_1} - \text{H.c.}\right)|\pm\rangle \\ &= \frac{i}{\sqrt{2}}\left(|1_{\boldsymbol{v}_1} 1_{\boldsymbol{v}_2} \ldots 1_{\boldsymbol{v}_m} 0_{\boldsymbol{o}_1} 0_{\boldsymbol{o}_2} \ldots 0_{\boldsymbol{o}_m}\rangle \mp i\,|0_{\boldsymbol{v}_1} 0_{\boldsymbol{v}_2} \ldots 0_{\boldsymbol{v}_m} 1_{\boldsymbol{o}_1} 1_{\boldsymbol{o}_2} \ldots 1_{\boldsymbol{o}_m}\rangle\right) \\ &= \pm\frac{1}{\sqrt{2}}\left(|0_{\boldsymbol{v}_1} 0_{\boldsymbol{v}_2} \ldots 0_{\boldsymbol{v}_m} 1_{\boldsymbol{o}_1} 1_{\boldsymbol{o}_2} \ldots 1_{\boldsymbol{o}_m}\rangle \pm i\,|1_{\boldsymbol{v}_1} 1_{\boldsymbol{v}_2} \ldots 1_{\boldsymbol{v}_m} 0_{\boldsymbol{o}_1} 0_{\boldsymbol{o}_2} \ldots 0_{\boldsymbol{o}_m}\rangle\right) \\ &= \pm 1\,|\pm\rangle \end{aligned} \tag{S12}$$

One can easily verify that a quantum state $|\psi\rangle$ that is an arbitrary superposition of any basis states apart from $|\pm\rangle$ gives rise to $G\,|\psi\rangle = 0\,|\psi\rangle$. Consequently, for the generator G of an m-electron excitation, we find two unique eigenstates $|\pm\rangle$ corresponding to the eigenvalues $\omega_\pm = \pm 1$, as well as a $(4^m - 2)$-dimensional eigenspace corresponding to the eigenvalue $\omega = 0$. All results hold equivalently for qubit-excitations. □

Last, we provide a third proof, which shows how our results can be unified with the SMO method [24]. The idea is mostly based on the work from Ref. [25].

Proof (alternative II). We once again assume a Hermitian generator with the property $G^3 = G$. The generator is then decomposed into the sum of two commuting self-inverse generators $G_\pm$, that is

$$G = \frac{1}{2}\left(G_+ + G_-\right), \tag{S13}$$

where

$$G_\pm := G \pm (G^2 - 1). \tag{S14}$$

We first verify that $G_\pm$ are indeed self-inverse:

$$\begin{aligned} G_\pm^2 &= \left[G \pm (G^2-1)\right]^2 \\ &= G^2 \pm 2G(G^2-1) + (G^2-1)^2 \\ &= G^2 \pm 2\underbrace{(G^3 - G)}_{=0} + \underbrace{G^4}_{=G^2} - 2G^2 + 1 \\ &= 1. \end{aligned} \tag{S15}$$

The commutation of G_+ and G_- is a trivial result, since any operator commutes with any power of itself. The unitary $U(\theta) = \exp(-i\theta G)$ can thus be exactly decomposed as

$$U(\theta) = U_-(\theta)U_+(\theta) = \exp\left(-\frac{i}{2}\theta G_-\right)\exp\left(-\frac{i}{2}\theta G_+\right) \tag{S16}$$

According to the SMO case describing multiple occurrences of the same parameter (c.f. Eq. (S37)), the energy landscape of any observable varied by an operation assuming the form in Eq. (S16) gives rise to a second-order Fourier series.

□

Supplementary Note 4.2: $G^3 = G$ and $G^2 \neq I$ for generators of excitation operators

In this section, we derive that fermionic- and qubit-excitation generators fulfill the property $G^3 = G$, which is the foundation of ExcitationSolve. We start from the m-electron excitation generators introduced in the "Supported types of excitation operators" subsection in the "ExcitationSolve algorithm" subsection in the Results of the main text, namely:

$$\tau_{\boldsymbol{o},\boldsymbol{v}}^{(m)} = \prod_{l=1}^{m} a_{v_l}^\dagger a_{o_l} - \text{H.c.}, \tag{Eq. (4) revisited}$$

where the fermionic creation and annihilation operators obey the canonical anti-commutation relations $\{a_i, a_j^\dagger\} = \delta_{ij}$ and $\{a_i^\dagger, a_j^\dagger\} = \{a_i, a_j\} = 0$. For the second power of $\tau_{\boldsymbol{o},\boldsymbol{v}}^{(m)}$, we obtain

$$\tau_{\boldsymbol{o},\boldsymbol{v}}^{(m)^2} = \prod_{l=1}^{m} a_{v_l}^\dagger a_{o_l} a_{v_l}^\dagger a_{o_l} + \prod_{l=1}^{m} a_{o_l}^\dagger a_{v_l} a_{o_l}^\dagger a_{v_l} - \prod_{l=1}^{m} a_{v_l}^\dagger a_{o_l} a_{o_l}^\dagger a_{v_l} - \prod_{l=1}^{m} a_{o_l}^\dagger a_{v_l} a_{v_l}^\dagger a_{o_l}. \tag{S17}$$

A direct implication of the anti-commutation relations is that $a_i^{\dagger 2} = a_i^2 = 0$ and $[a_i^{(\dagger)}, a_j a_j^\dagger] = 0$. Using this we find that

$$\tau_{\boldsymbol{o},\boldsymbol{v}}^{(m)^2} = -\left(\prod_{l=1}^{m} a_{v_l}^\dagger a_{v_l} a_{o_l} a_{o_l}^\dagger + \text{H.c.}\right), \tag{S18}$$

which clearly is not an identity operator. Next, for the third power, we similarly obtain from Eq. (4) and (S18) that

$$\tau_{\boldsymbol{o},\boldsymbol{v}}^{(m)^3} = -\left(\prod_{l=1}^{m} a_{v_l}^\dagger a_{v_l} a_{v_l}^\dagger a_{o_l} a_{o_l}^\dagger a_{o_l} - \text{H.c.}\right). \tag{S19}$$

Utilizing that $a_i^\dagger a_i a_i^\dagger = a_i^\dagger(1 - a_i^\dagger a_i) = a_i^\dagger$ and similarly $a_i a_i^\dagger a_i = a_i$, we finally arrive at

$$\tau_{\boldsymbol{o},\boldsymbol{v}}^{(m)^3} = -\left(\prod_{l=1}^{m} a_{v_l}^\dagger a_{o_l} - \text{H.c.}\right) = -\tau_{\boldsymbol{o},\boldsymbol{v}}^{(m)}. \tag{S20}$$

Now we are presented with an anti-Hermitian operator of the form $G^3 = -G$ generating the excitation operator $\exp(\theta G)$. To fit it within the convention of writing gates in terms of their Hermitian generator, we redefine $G := i\tau_{\boldsymbol{o},\boldsymbol{v}}^{(m)}$ and therefore obtain $G^3 = G$. This logic can also be easily inferred from the following equation:

$$U_{\boldsymbol{o},\boldsymbol{v}}^{(m)}(\theta) = \exp(\theta\ \tau_{\boldsymbol{o},\boldsymbol{v}}^{(m)}) = \exp(-i^2\theta\ \tau_{\boldsymbol{o},\boldsymbol{v}}^{(m)}) = \exp(-i\theta\ \underbrace{i\tau_{\boldsymbol{o},\boldsymbol{v}}^{(m)}}_{=G}). \tag{S21}$$

The same properties can easily be shown for qubit-excitation generators. In QEB-ansätze, the fermionic creation and annihilation operators $a^\dagger$ and a in Eq. (4) are replaced by qubit creation- and annihilation operators $Q^\dagger = \sigma^-$ and $Q = \sigma^+$ [26], giving rise to the qubit-excitation generator. These operators fulfill the commutation relations $[Q_i, Q_j^\dagger] = \delta_{ij}(1 - 2Q_i^\dagger Q_i)$ with the (qubit-) occupation number $n_i = Q_i^\dagger Q_i$ being restricted to 0 or 1. These are the same algebraic properties as known for hard-core bosons [27] or parafermions [28], allowing for a mapping-independent interpretation of qubit-excitations[2]. The steps of the proof are the same, apart from skipping the sign argument due to the non-local commutation relations between qubit-creation/annihilation operators.

Supplementary Note 4.3: General Fourier Series for Multi-Parameter Optimization

In Supplementary Note 4.1, we have derived an analytical expression for the energy functional in a single parameter, which takes the form of a second-order Fourier series (c.f. Eq. (3)). In the following, we prove inductively that an D-dimensional multi-parameter optimization landscape assumes the form of a D-dimensional second-order Fourier series.

Proof. The base case, that is $D = 1$, has already been proven in Supplementary Note 4.1. For the induction step, we assume that, without loss of generality, the $(D+1)$-th parameter acts on the quantum state after the previous D parameters. We define the ordered index sets $\mathcal{M}^{(D)} = \{j, \ldots, k\}$, which contains the D simultaneously optimized parameters in ascending order, and $\mathcal{M}^{(D+1)} = \{j, \ldots, k, l\}$, which further includes the index l of the $(D+1)$-th parameter θ_l. To establish the induction hypothesis and carry out the induction step, we first define the effective initial state

$$|\psi'\rangle := \left(\prod_{i<j} U(\theta_i)\right) |\psi_0\rangle\,, \qquad \text{(Eq. (S4) revisited)}$$

and the effective Hamiltonian

$$H^{(D)} := \left(\prod_{i>\max\mathcal{M}^{(D)}} U(\theta_i)\right)^\dagger H \left(\prod_{i>\max\mathcal{M}^{(D)}} U(\theta_i)\right). \tag{S22}$$

[2]In the literature, the generated qubit-excitation gates are also sometimes referred to as Givens rotations [29, 30], as they can be visualized as a rotation in a two-dimensional subspace. More details about this subspace can be inferred from Eq. (S11) in Supplementary Note 4.1.

We further denote the effective unitary, including all operations sandwiched by the first and last variational (not fixed) unitary, for D parameters as

$$U^{(D)} := \prod_{\substack{i \geq \min \mathcal{M}^{(D)} \\ i \leq \max \mathcal{M}^{(D)}}} U(\theta_i). \tag{S23}$$

Following these definitions, we may express the induction hypothesis as

$$f_{\boldsymbol{\theta}}(\boldsymbol{\theta}_{\mathcal{M}^{(D)}}) = \langle\psi'| \, U^{\dagger(D)} H^{(D)} U^{(D)} \, |\psi'\rangle = \boldsymbol{c}^{(D)} \cdot \left[\bigotimes_{i \in \mathcal{M}^{(D)}} \begin{pmatrix} \cos(\theta_i) \\ \cos(2\theta_i) \\ \sin(\theta_i) \\ \sin(2\theta_i) \\ 1 \end{pmatrix} \right], \tag{S24}$$

where $H^{(D)}$ is some arbitrary Hamiltonian since H is arbitrary. Next, we abbreviate all the fixed operations between k and l as

$$V^{(D+1)} := \prod_{\substack{i > \max \mathcal{M}^{(D)} \\ i < \max \mathcal{M}^{(D+1)}}} U(\theta_i). \tag{S25}$$

The energy landscape of the $(D+1)$-parameter case can then be written as

$$\begin{aligned} f_{\boldsymbol{\theta}}(\boldsymbol{\theta}_{\mathcal{M}^{(D+1)}}) &= \langle\psi'|U^{\dagger(D+1)} H^{(D+1)} U^{(D+1)}|\psi'\rangle & \text{(S26)} \\ &= \langle\psi'|U^{\dagger(D)} V^{\dagger(D+1)} U^{\dagger}(\theta_l) H^{(D+1)} U(\theta_l) V^{(D+1)} U^{(D)}|\psi'\rangle & \text{(S27)} \end{aligned}$$

Using the same reasoning as in Supplementary Note 4.1, that is the Euler formula in Eq. S3 and the trigonometric identities, we find that

$$\begin{aligned} f_{\boldsymbol{\theta}}(\boldsymbol{\theta}_{\mathcal{M}^{(D+1)}}) = \quad & \langle\psi'| \, U^{\dagger(D)} \underbrace{V^{\dagger} \left(\left\{H, G^2\right\} - 2G^2 H G^2\right) V}_{H_1} U^{(D)} \, |\psi'\rangle \cos(\theta_l) \\ & + \tfrac{1}{2} \langle\psi'| \, U^{\dagger(D)} \underbrace{V^{\dagger} \left(G^2 H G^2 - GHG\right) V}_{H_2} U^{(D)} \, |\psi'\rangle \cos(2\theta_l) \\ & + \langle\psi'| \, U^{\dagger(D)} \underbrace{V^{\dagger} \left(i \, [G, H] - i \, [GHG, G]\right) V}_{H_3} U^{(D)} \, |\psi'\rangle \sin(\theta_l) \\ & + \tfrac{1}{2} \langle\psi'| \, U^{\dagger(D)} \underbrace{V^{\dagger} i \, [GHG, G] \, V}_{H_4} U^{(D)} \, |\psi'\rangle \sin(2\theta_l) \\ & + \langle\psi'| \, U^{\dagger(D)} \underbrace{V^{\dagger} \left(H - \left\{H, G^2\right\} + GHG + 3G^2 H G^2\right) V}_{H_5} U^{(D)} \, |\psi'\rangle \, , \end{aligned} \tag{S28}$$

where we abbreviated $H^{(D+1)} = H$ and $V^{(D+1)} = V$. Notice that all of the expectation values $\langle\psi'| \, U^{\dagger(D)} H_i U^{(D)} \, |\psi'\rangle$ for $i = 1, \ldots, 5$ must assume a D-dimensional second-order Fourier series according to the induction hypothesis in Eq. (S24) (the coefficients $\boldsymbol{c}^{(D)}$ differ across the different effective Hamiltonians H_i). Finally, we conclude that the energy landscape can be rewritten as

$$f_{\boldsymbol{\theta}}(\boldsymbol{\theta}_{\mathcal{M}^{(D+1)}}) = \boldsymbol{c}^{(D+1)} \cdot \left[\bigotimes_{i \in \mathcal{M}^{(D+1)}} \begin{pmatrix} \cos(\theta_i) \\ \cos(2\theta_i) \\ \sin(\theta_i) \\ \sin(2\theta_i) \\ 1 \end{pmatrix} \right], \tag{S29}$$

thus completing the proof.

□

Supplementary Note 4.4: Fourier series for multiple occurrences of a single parameter

In this Supplementary Note, we derive an expression for the energy functional in a single parameter which occurs S times in the circuit. We will inductively prove that the energy landscape is given by finite Fourier series of order $2S$:

$$f_{\boldsymbol{\theta}}(\theta) = \sum_{s=1}^{2S} a_s \cos(s\theta_j) + \sum_{s=1}^{2S} b_s \sin(s\theta_j) + c. \qquad \text{(Eq. (9) revisited)}$$

Proof. Once again, the base case $S = 1$ has already been proved in Supplementary Note 4.1. Assuming that Eq. (9) holds for some S, we consider the case with $S+1$ occurrences. Following exactly the same steps as in the previous proof of the multi-parameter case (Supplementary Note 4.3), but redefining $\mathcal{M}^{(S)}$ such that it corresponds to the equal parameters, i.e. $\boldsymbol{\theta}_{\mathcal{M}^{(S)}} = \theta$, we find that the energy landscape is given by

$$f_{\boldsymbol{\theta}}^{(S+1)}(\theta) = a_1^{(s)}(\theta)\cos(\theta) + a_2^{(s)}(\theta)\cos(2\theta) + b_1^{(s)}(\theta)\sin(\theta) + b_2^{(s)}(\theta)\sin(2\theta) + c^{(s)}(\theta), \tag{S30}$$

where the parameterized coefficients $a_1^{(s)}(\theta), a_2^{(s)}(\theta), b_1^{(s)}(\theta), b_2^{(s)}(\theta)$ and $c^{(s)}(\theta)$ obey the induction assumption from Eq. (9). To obtain the order of the Fourier series, we need to compute the highest possible frequency $\omega_{\max}^{(S+1)}$ obtained from reducing the trigonometric form of the expressions above. For that purpose, we employ the following trigonometric identities:

$$\begin{aligned}
\sin(ax)\sin(bx) &= \frac{1}{2}\left[\cos((a-b)x) - \cos((a+b)x)\right], \\
\sin(ax)\cos(bx) &= \frac{1}{2}\left[\sin((a-b)x) + \sin((a+b)x)\right], \\
\cos(ax)\cos(bx) &= \frac{1}{2}\left[\cos((a-b)x) + \cos((a+b)x)\right].
\end{aligned} \tag{S31}$$

The highest frequency is thus obtained as the sum of the largest frequency for S occurrences, i.e. $\omega_{\max}^{(S)} = 2S$, and the additional double frequency 2 of the $(S+1)$-th occurrence, giving rise to $\omega_{\max}^{(S+1)} = 2(S+1)$, and thus

$$f_{\boldsymbol{\theta}}^{(S+1)}(\theta) = \sum_{s=1}^{2(S+1)} a_s \cos(s\theta_j) + \sum_{s=1}^{2(S+1)} b_s \sin(s\theta_j) + c. \tag{S32}$$

□

Supplementary Note 5: Comprehensive overview of standard approaches in variational quantum algorithms

Supplementary Note 5.1: Gradients via parameter-shift rules for excitation operators

For gradient-based optimization of parameterized quantum circuits, analytical gradients can be computed for specific types of parameterized operators and gates through so-called *parameter-shift rules.* As the name suggests, the (partial) derivative of a function $f_{\boldsymbol{\theta}}$ w.r.t. parameter θ_j is composed of energy function evaluations at shifts of parameter θ_j. For excitation operators, fulfilling the generator property $G^3 = G$ without being self-inverse, i.e., $G^2 \neq I$, Ref. [18] states the four-term paramter-shift rule relying on the energy values of four parameter shifts $\pm\alpha, \pm\beta$ as

$$f'_{\boldsymbol{\theta}}(\theta_j) = \frac{\partial}{\partial\theta_j} f(\boldsymbol{\theta}) = d_1\left(f_{\boldsymbol{\theta}}(\theta_j+\alpha) - f_{\boldsymbol{\theta}}(\theta_j-\alpha)\right) - d_2\left(f_{\boldsymbol{\theta}}(\theta_j+\beta) - f_{\boldsymbol{\theta}}(\theta_j-\beta)\right) \tag{S33}$$

with, for example,

$$d_1 = \tfrac{1}{2}, \qquad d_2 = \tfrac{\sqrt{2}-1}{4}, \qquad \alpha = \tfrac{\pi}{2}, \qquad \beta = \pi. \tag{S34}$$

Other choices of $\alpha, \beta, d_{1,2}$ are possible subject to conditions [18].

Variations of parameter-shift rules exist in which the quantum circuit is dressed by additional gates. This leads to a decrease in the number of required shifts and, hence, energy evaluations on the quantum device to *two* if the wave function (i.e., quantum state) is *real.* For excitation operators, this was derived in Ref. [25]. As both the four-term parameter-shift rule [18] and ExcitationSolve rely on energies of pure parameter shifts, the four-term parameter-shift rule is considered for a fair comparison between ExcitationSolve and gradient-based optimizers. While this approach requires circuits differing from the ones necessary to evaluate the energy, the cost of the distinct circuit component may be upper-bounded by the cost of a regular excitation [25]. Therefore, we may for the sake of simplicity still compare the costs in terms of energy evaluations, even though the shifts for the real wave functions are strictly speaking not energy evaluations as in ExcitationSolve or other optimizers (e.g. Table 1).

Supplementary Note 5.2: Quantum-aware optimization for rotations: Rotosolve and SMO

The Rotosolve [31] optimization method describes a coordinate descent approach, i.e., only a single parameter θ_j is updated in each iteration while the other parameters $\theta_{i\neq j}$ are held fixed. Multiple extensions of Rotosolve/SMO have been proposed such as Free-Axis Selection (Fraxis) [32–34], Free Quaternion Selection (FQS) [35,36] and the Unitary Block Optimization Scheme (UBOS) [37]. On the other hand, Rotosolve has never been extended to excitation operators as studied here despite several attempts [38,39] using polynomial fits, unaware of the correct analytical form as a second-order Fourier series. For each update step, the entire energy function along the current parameter is reconstructed, which has the form of a simple cosine curve

$$f_{\boldsymbol{\theta}}(\theta_j) = A\cos(\theta_j - \Phi) + c, \tag{S35}$$

and the parameter is set to the then classically and analytically determined minimum of the reconstruction. To determine the coefficients A, Φ, c the energy is evaluated on the quantum computer for three suitable shifts of the parameter θ_j. Note that one evaluation can be saved by reusing the energy value from the previous iteration. Importantly, the applicability of Rotosolve is limited to rotations, i.e., parameterized operators of the form $\exp(i\theta_j G/2)$ with $G^2 = I$, and, moreover, all parameters must be independent of each other, meaning that each θ_j must only occur once in the variational quantum circuit.

While Rotosolve was independently proposed under the name Sequential Minimal Optimization (SMO) in the first variant in Ref. [24], SMO comes in two further variants: The second variant of SMO is a multi-parameter generalization, concerning the simultaneous optimization of a *subset* of parameters $\boldsymbol{\theta}_{\mathcal{M}}$ where $\mathcal{M}$ denotes the index set of the $|\mathcal{M}| = D$ parameters to be optimized. A

multi-parameter generalization was also mentioned in Ref. [40]. The D-dimensional energy function reconstruction includes 3^D coefficients $\boldsymbol{c}$ and has the analytical form of

$$f_{\boldsymbol{\theta}}(\boldsymbol{\theta}_{\mathcal{M}}) = \boldsymbol{c} \cdot \left[\bigotimes_{i \in \mathcal{M}} \begin{pmatrix} \cos(\theta_i) \\ \sin(\theta_i) \\ 1 \end{pmatrix} \right]. \tag{S36}$$

The third variant of SMO lifts the requirement that each parameter must occur once in the variational quantum circuit, which SMO and Rotosolve impose otherwise. If a parameter θ_j occurs S times, the energy function along parameter θ_j is no longer a simple cosine function but incorporates S frequencies, i.e., obeys the form of a Fourier series of order S as

$$f_{\boldsymbol{\theta}}(\theta_j) = \sum_{s=1}^{S} a_s \cos(s\theta_j) + \sum_{s=1}^{S} b_s \sin(s\theta_j) + c. \tag{S37}$$

Thus, determining $2S+1$ coefficients require $2S+1$ energy evaluations on the quantum computer to obtain the reconstruction to optimize θ_j (Again, one evaluation can be skipped by reusing the final energy of the previous iteration.)

Supplementary Note 5.3: ADAPT-VQE

In ADAPT-VQE, we optimize adaptive ansätze in VQE by alternately growing of the ansatz and optimization of the parameters as introduced in Ref. [41]. Each Adapt(VQE)-Step consists of two parts: First, a suitable operator is appended to the ansatz from an operator pool, e.g., the pool of all single and double fermionic excitation operators. Second, all parameters are re-optimized while keeping the ansatz fixed, which equals a standard VQE run with a warm-start, i.e., the parameter values from the previous Adapt-Steps are used as initial guesses (while the newly added operator is initialized with its parameter set to zero.)

For selecting a new operator from the pool, a scoring criterion assesses the quality of each operator candidate. The original ADAPT-VQE [41] obeys a gradient-based criterion where the operator is selected that admits the highest magnitude of its partial derivative in zero. This is the operator with the strongest *local* impact on the energy. While the partial derivative could be computed through the (four-term) parameter-shift rule as in Eq. (S33), the partial derivative in zero constitutes a special case such that it can be alternatively expressed through the expectation value of a commutator

$$\left.\frac{\partial f(\theta_1, \theta_2, \ldots, \theta_N, \theta_{N+1})}{\partial \theta_{N+1}}\right|_{\theta_{N+1}=0} = \langle i\,[H, G] \rangle \tag{S38}$$

where H and G are the Hamiltonian and generator of the tested excitation operator as in Eq. (2), respectively. The expectation is taken over the state $|\psi^{(N)}\rangle$, which is prepared by the previous N parameters in the current ansatz before being extended.

Supplementary References

[1] Ville Bergholm, Josh Izaac, Maria Schuld, Christian Gogolin, Shahnawaz Ahmed, Vishnu Ajith, M. Sohaib Alam, Guillermo Alonso-Linaje, B. AkashNarayanan, Ali Asadi, Juan Miguel Arrazola, Utkarsh Azad, Sam Banning, Carsten Blank, Thomas R Bromley, Benjamin A. Cordier, Jack Ceroni, Alain Delgado, Olivia Di Matteo, Amintor Dusko, Tanya Garg, Diego Guala, Anthony Hayes, Ryan Hill, Aroosa Ijaz, Theodor Isacsson, David Ittah, Soran Jahangiri, Prateek Jain, Edward Jiang, Ankit Khandelwal, Korbinian Kottmann, Robert A. Lang, Christina Lee, Thomas Loke, Angus Lowe, Keri McKiernan, Johannes Jakob Meyer, J. A. Montañez-Barrera, Romain Moyard, Zeyue Niu, Lee James O'Riordan, Steven Oud, Ashish Panigrahi, Chae-Yeun Park, Daniel Polatajko, Nicolás Quesada, Chase Roberts, Nahum Sá, Isidor Schoch, Borun Shi, Shuli Shu, Sukin Sim, Arshpreet Singh, Ingrid Strandberg, Jay Soni, Antal Száva, Slimane Thabet, Rodrigo A. Vargas-Hernández, Trevor Vincent, Nicola Vitucci, Maurice Weber, David Wierichs, Roeland Wiersema, Moritz Willmann, Vincent Wong, Shaoming Zhang, and Nathan Killoran. Pennylane: Automatic differentiation of hybrid quantum-classical computations, 2022.

[2] Abhinav Kandala, Antonio Mezzacapo, Kristan Temme, Maika Takita, Markus Brink, Jerry M. Chow, and Jay M. Gambetta. Hardware-efficient variational quantum eigensolver for small molecules and quantum magnets. *Nature*, 549(7671):242–246, September 2017.

[3] James C. Spall. An overview of the simultaneous perturbation method for efficient optimization. *Hopkins APL Technical Digest*, 19(4):482–492, 1998.

[4] M.J. Powell. A direct search optimization method that models the objective and constraint functions by linear interpolation. *Advances in Optimization and Numerical Analysis*, pages 51–67, 1994.

[5] Utkarsh Azad. Pennylane quantum chemistry datasets. https://pennylane.ai/datasets/qchem/h2-molecule, https://pennylane.ai/datasets/qchem/h3-plus-molecule, https://pennylane.ai/datasets/qchem/lih-molecule, https://pennylane.ai/datasets/qchem/h2o-molecule, 2023.

[6] Pascual Jordan and Eugene Paul Wigner. *Über das paulische äquivalenzverbot*. Springer, 1993.

[7] Youngseok Kim, Christopher J. Wood, Theodore J. Yoder, Seth T. Merkel, Jay M. Gambetta, Kristan Temme, and Abhinav Kandala. Scalable error mitigation for noisy quantum circuits produces competitive expectation values. *Nature Physics*, 19(5):752–759, May 2023.

[8] David C. McKay, Christopher J. Wood, Sarah Sheldon, Jerry M. Chow, and Jay M. Gambetta. Efficient z gates for quantum computing. *Physical Review A*, 96(2):022330, August 2017.

[9] Sergey Bravyi, Jay M. Gambetta, Antonio Mezzacapo, and Kristan Temme. Tapering off qubits to simulate fermionic Hamiltonians, January 2017.

[10] Kanav Setia, Richard Chen, Julia E. Rice, Antonio Mezzacapo, Marco Pistoia, and James D. Whitfield. Reducing Qubit Requirements for Quantum Simulations Using Molecular Point Group Symmetries. *Journal of Chemical Theory and Computation*, 16(10):6091–6097, October 2020.

[11] John Shawe-Taylor and Nello Cristianini. *Kernel Methods for Pattern Analysis*. Cambridge University Press, Cambridge, 2004.

[12] Kevin P. Murphy. *Probabilistic Machine Learning: An Introduction*. MIT Press, 2022.

[13] Michael A. Nielsen and Isaac L. Chuang. *Quantum Computation and Quantum Information*. Cambridge University Press, Cambridge ; New York, 10th anniversary ed edition, 2010.

[14] Youngkyu Sung, Félix Beaudoin, Leigh M. Norris, Fei Yan, David K. Kim, Jack Y. Qiu, Uwe von Lüpke, Jonilyn L. Yoder, Terry P. Orlando, Simon Gustavsson, Lorenza Viola, and William D.

Oliver. Non-Gaussian noise spectroscopy with a superconducting qubit sensor. *Nature Communications*, 10(1):3715, September 2019.

[15] Katsuhiro Endo, Yuki Sato, Rudy Raymond, Kaito Wada, Naoki Yamamoto, and Hiroshi C. Watanabe. Optimal parameter configurations for sequential optimization of the variational quantum eigensolver. *Physical Review Research*, 5(4):043136, November 2023.

[16] Elias M. Stein and Rami Shakarchi. *Fourier Analysis: An Introduction*. Number 1 in Princeton Lectures in Analysis / Elias M. Stein & Rami Shakarchi. Princeton University Press, Princeton Oxford, 15. druck edition, 2003.

[17] Trevor Hastie, Robert Tibshirani, Jerome H Friedman, and Jerome H Friedman. *The Elements of Statistical Learning: Data Mining, Inference, and Prediction*, volume 2. Springer, 2009.

[18] Gian-Luca R Anselmetti, David Wierichs, Christian Gogolin, and Robert M Parrish. Local, expressive, quantum-number-preserving VQE ansätze for fermionic systems. *New Journal of Physics*, 23(11):113010, November 2021.

[19] Francesco A Evangelista, Garnet Kin Chan, and Gustavo E Scuseria. Exact parameterization of fermionic wave functions via unitary coupled cluster theory. *The Journal of chemical physics*, 151(24), 2019.

[20] Luogen Xu, Joseph T Lee, and JK Freericks. Test of the unitary coupled-cluster variational quantum eigensolver for a simple strongly correlated condensed-matter system. *Modern Physics Letters B*, 34(19n20):2040049, 2020.

[21] Jia Chen, Hai-Ping Cheng, and James K Freericks. Quantum-inspired algorithm for the factorized form of unitary coupled cluster theory. *Journal of Chemical Theory and Computation*, 17(2):841–847, 2021.

[22] James K Freericks. Operator relationship between conventional coupled cluster and unitary coupled cluster. *Symmetry*, 14(3):494, 2022.

[23] David Wierichs, Josh Izaac, Cody Wang, and Cedric Yen-Yu Lin. General parameter-shift rules for quantum gradients. *Quantum*, 6:677, March 2022.

[24] Ken M. Nakanishi, Keisuke Fujii, and Synge Todo. Sequential minimal optimization for quantum-classical hybrid algorithms. *Physical Review Research*, 2(4):043158, October 2020.

[25] Jakob S. Kottmann, Abhinav Anand, and Alán Aspuru-Guzik. A feasible approach for automatically differentiable unitary coupled-cluster on quantum computers. *Chemical Science*, 12(10):3497–3508, March 2021.

[26] Yordan S. Yordanov, V. Armaos, Crispin H. W. Barnes, and David R. M. Arvidsson-Shukur. Qubit-excitation-based adaptive variational quantum eigensolver. *Communications Physics*, 4(1):1–11, October 2021.

[27] Jacob Jordan, Román Orús, and Guifré Vidal. Numerical study of the hard-core bose-hubbard model on an infinite square lattice. *Phys. Rev. B*, 79:174515, May 2009.

[28] L-A Wu and DA Lidar. Qubits as parafermions. *Journal of Mathematical Physics*, 43(9):4506–4525, 2002.

[29] Juan Miguel Arrazola, Olivia Di Matteo, Nicolás Quesada, Soran Jahangiri, Alain Delgado, and Nathan Killoran. Universal quantum circuits for quantum chemistry. *Quantum*, 6:742, June 2022.

[30] Kaelyn J. Ferris, A. J. Rasmusson, Nicholas T. Bronn, and Olivia Lanes. Quantum simulation on noisy superconducting quantum computers, 2022.

[31] Mateusz Ostaszewski, Edward Grant, and Marcello Benedetti. Structure optimization for parameterized quantum circuits. *Quantum*, 5:391, 2021.

[32] Hiroshi C. Watanabe, Rudy Raymond, Yu-Ya Ohnishi, Eriko Kaminishi, and Michihiko Sugawara. Optimizing Parameterized Quantum Circuits with Free-Axis Selection. In *2021 IEEE International Conference on Quantum Computing and Engineering (QCE)*, pages 100–111, October 2021.

[33] Hiroshi C. Watanabe, Rudy Raymond, Yu-Ya Ohnishi, Eriko Kaminishi, and Michihiko Sugawara. Optimizing Parameterized Quantum Circuits With Free-Axis Single-Qubit Gates. *IEEE Transactions on Quantum Engineering*, 4:1–16, 2023.

[34] Kaito Wada, Rudy Raymond, Yu-ya Ohnishi, Eriko Kaminishi, Michihiko Sugawara, Naoki Yamamoto, and Hiroshi C. Watanabe. Simulating time evolution with fully optimized single-qubit gates on parametrized quantum circuits. *Physical Review A*, 105(6):062421, June 2022.

[35] Kaito Wada, Rudy Raymond, Yuki Sato, and Hiroshi C. Watanabe. Sequential optimal selections of single-qubit gates in parameterized quantum circuits. *Quantum Science and Technology*, 9(3):035030, May 2024.

[36] Hiroyoshi Kurogi, Katsuhiro Endo, Yuki Sato, Michihiko Sugawara, Kaito Wada, Kenji Sugisaki, Shu Kanno, Hiroshi C. Watanabe, and Haruyuki Nakano. Optimizing a parameterized controlled gate with Free Quaternion Selection, 2024.

[37] Lucas Slattery, Benjamin Villalonga, and Bryan K. Clark. Unitary block optimization for variational quantum algorithms. *Physical Review Research*, 4(2):023072, April 2022.

[38] V. Armaos, Dimitrios A. Badounas, Paraskevas Deligiannis, Konstantinos Lianos, and Yordan S. Yordanov. Efficient Parabolic Optimisation Algorithm for adaptive VQE implementations, October 2021.

[39] Weitang Li, Yufei Ge, Shixin Zhang, Yuqin Chen, and Shengyu Zhang. Efficient and Robust Parameter Optimization of the Unitary Coupled-Cluster Ansatz, January 2024.

[40] Robert M. Parrish, Joseph T. Iosue, Asier Ozaeta, and Peter L. McMahon. A Jacobi Diagonalization and Anderson Acceleration Algorithm For Variational Quantum Algorithm Parameter Optimization, April 2019.

[41] Harper R. Grimsley, Sophia E. Economou, Edwin Barnes, and Nicholas J. Mayhall. An adaptive variational algorithm for exact molecular simulations on a quantum computer. *Nature Communications*, 10(1):3007, July 2019.
